# Correlation between *EGFR* Amplification and the Expression of MicroRNA-200c in Primary Glioblastoma Multiforme

**DOI:** 10.1371/journal.pone.0102927

**Published:** 2014-07-24

**Authors:** Eva Serna, Concha Lopez-Gines, Daniel Monleon, Lisandra Muñoz-Hidalgo, Robert C. Callaghan, Rosario Gil-Benso, Horacio Martinetto, Aurelia Gregori-Romero, Jose Gonzalez-Darder, Miguel Cerda-Nicolas

**Affiliations:** 1 Departamento de Patología, Universitat de Valencia, Valencia, Spain; 2 Unidad Central de Investigación en Medicina, Universitat de València, Valencia, Spain; 3 Fundación de Investigación del Hospital Clínico Universitario de Valencia/INCLIVA, Valencia, Spain; 4 Department of Neuropathology, Institute for Neurological Research, FLENI, Buenos Aires, Argentina; 5 Servicio de Neurocirugía, Hospital Clínico Universitario de Valencia, Valencia, Spain; Swedish Medical Center, United States of America

## Abstract

Extensive infiltration of the surrounding healthy brain tissue is a critical feature in glioblastoma. Several miRNAs have been related to gliomagenesis, some of them related with the EGFR pathway. We have evaluated whole-genome miRNA expression profiling associated with different *EGFR* amplification patterns, studied by fluorescence *in situ* hybridization in tissue microarrays, of 30 cases of primary glioblastoma multiforme, whose clinicopathological and immunohistochemical features have also been analyzed. MicroRNA-200c showed a very significant difference between tumors having or not *EGFR* amplification. This microRNA plays an important role in epithelial-mesenchymal transition, but its implication in the behavior of glioblastoma is largely unknown. With respect to *EGFR* status our cases were categorized into three groups: high level *EGFR* amplification, low level *EGFR* amplification, and no *EGFR* amplification. Our results showed that microRNA-200c and E-cadherin expression are down-regulated, while ZEB1 is up-regulated, when tumors showed a high level of *EGFR* amplification. Conversely, ZEB1 mRNA expression levels were significantly lower in the group of tumors without *EGFR* amplification. Tumors with a low level of *EGFR* amplification showed *ZEB1* expression levels comparable to those detected in the group with a high level of amplification. In this study we provide what is to our knowledge the first report of association between microRNA-200c and *EGFR* amplification in glioblastomas.

## Introduction

Glioblastoma multiforme (GBM) is the most common and most aggressive malignant primary brain tumor in humans. The majority of GBMs arise *de novo* and are defined as primary GBMs, while the progression from lower grade astrocytomas results in secondary GBMs. These tumors are characterized by an extremely poor prognosis and the patients have a median survival of approximately 14 months [1], [2]. The growth and invasive nature of GBM are promoted by dysregulation of multiple signaling pathways [3].

Epidermal growth factor receptor (*EGFR*) amplification, observed in about 35–70% of GBMs, constitutes a lesion signature for these tumors [4]–[6]. According to the *EGFR* status, gene copy number and type of amplification, tumors have been categorized into three groups [7]: GBM with high level of *EGFR* amplification as double minutes (dmin), in which the fraction of cells with amplification is higher than 20% and with more than 25 EGFR signals per cell, GBM with low level of *EGFR* amplification as insertions into different loci on chromosome 7, with a 5–20% of cells with amplification and less than 20 EGFR signals per cell, and GBM without *EGFR* amplification.

The activation, over-expression and amplification of *EGFR* are associated with oncogenesis in GBMs. EGFR protein over-expression is usually associated with gene amplification in GBMs and these two parameters have been proposed as potential prognostic indicators, although their validity as accurate prognostic factors in these tumors is unclear [8], [9].

MicroRNA (miRNA) is a type of non-coding RNA of approximately 20–30 nucleotides in length that has been identified as an important regulator of mRNA translation [10], [11]. miRNAs regulate diverse cellular processes and some of them have been shown to function as either tumor suppressors or oncogenes [12], [13]. miRNAs are emerging as novel players in tumorigenesis. In this regard, miRNA expression is deregulated in most, if not all, types of cancer. Based on the literature the most common dysregulation of miRNAs in GBM is over-expression; more than 200 miRNAs have been found to be significantly over-expressed, among them miR-15b, miR-21, miR-221/222 and miR-296. Also, more than 90 miRNAs have been reported as down-regulated in GBM, compared to normal brain tissue. Of these, miR-7, miR-124, miR-128, miR-137 and miR-181a/b/c seem to have a predominant role in GBM pathogenesis [14]–[16].

In the present study we evaluated whole-genome miRNA expression profiling in samples of human GBMs associated with different *EGFR* amplification patterns. Of the miRNAs which show statistically significant associations with *EGFR* amplification we examined miR-200c in more detail. The miR-200 family is involved in the progression of different types of neoplasms and plays an important role in epithelial-mesenchymal transition (EMT) [17], [18]. A double-negative feedback loop between the repressor zinc-finger E-box binding homeobox (ZEB) family transcription factors and the miR-200 family has been shown to regulate EMT in different cell systems [19]–[21]. During EMT cells lose adhesion and increase their motility. One of the crucial steps promoting EMT is the repression of the epithelial marker E-cadherin, a cell membrane calcium-dependent glycoprotein responsible for cell-cell adhesion. ZEB family members (ZEB1 and ZEB2) are able to bind directly to the E-cadherin promoter and repress its transcription [19], [21].

To the best of our knowledge, the association of miR-200c with different levels of *EGFR* amplification has not been described in GBM. We hypothesize that the loss of miR-200c expression may be related to the *EGFR* amplification and could play a significant role in the GBM invasive phenotype.

## Materials and Methods

### Patient population and histopathological study

Tumor samples were obtained from 30 patients with primary GBM from the Department of Neurosurgery of the Hospital Clinico Universitario of Valencia. These tumors were considered as clinically primary GBMs, as they did not have a previous diagnosis of lower grade astrocytomas. This study was reviewed and approved by the Hospital Clinico Universitario of Valencia (CEIC) ethics committee. Patients gave written informed consent for participating in the study. During surgery, the resected tissue was sent for routine histological analysis, and the remainder was immediately put in cryogenic vials and snap-frozen in liquid nitrogen. All snap-frozen samples were stored in a freezer at −80°C until further analysis. All samples used for histopathological examination were fixed in neutral-buffered formalin, embedded in paraffin, sectioned and stained with hematoxylin-eosin. Tumors were classified according to the 2007 WHO Histological Classification [1] and diagnosed as glioblastoma multiforme.

The immunohistochemical study was performed on paraffin-embedded sections using the avidin–biotin peroxidase method. The study was carried out using antibodies against glial fibrillary acidic protein (GFAP; Dako, Glostrup, Denmark), Ki-67 (MIB-1, Dako) and monoclonal mouse anti-human EGFR (clone H11, Dako), which recognizes the wild-type EGFR and the deletion-mutant form of the receptor (EGFRvIII). Proliferation index was evaluated using MIB-1 antibody staining and was calculated by determining the percentage of immunopositive nuclei. EGFR expression was quantified according to the intensity of staining and number of stained cells as: 0 (no staining), 1 (light or focal), 2 (moderate) and 3 (strong). Scores of 0 or 1 were defined as no over-expression and scores of 2 and 3 as over-expression [8].

### Fluorescence In Situ Hybridization: *EGFR* amplification

GBM samples used for FISH analysis were studied using tissue microarrays (TMA). We removed four 0.6-mm cores, from paraffin blocks of each case, corresponding to tumoral areas, confirmed by hematoxylin-eosin staining, using the Beecher Instruments Manual Tissue ArrayerI (Beecher Instruments, Sun Prairie, WI, USA). FISH was carried out using the LSR EGFR Spectrum Orange/CEP 7 Spectrum Green Probe from Vysis (Abbott Laboratories, Downers Grove, IL, USA. Cat. No. 32-191053). Paraffin embedded TMAs were cut into 5-µm sections and these were mounted on Superfrost/Plus microscope slides (Microm International). Hybridizations were performed according to the manufacturer’s instructions. Counterstaining of nuclei was carried out using DAPI.

The fluorescent signal was detected using a photomicroscope, Leica DM400B, equipped with a set of the appropriate filters. Signals were counted in 100–150 non-overlapping tumor cell nuclei in the paraffin sections. The mean signal number for the EGFR gene and CEP 7 was calculated for each case, followed by the calculation of *EGFR*/CEP 7 ratio. The EGFR gene was quantified as amplified in individual cells when the *EGFR*/control signal ratio was greater than 2 [9].

### Multiplex Ligation-dependent Probe Amplification (MLPA): *EGFR* mutant form (*EGFRvIII*)

Biopsy punches from selected areas of paraffin blocks of each sample were used for DNA extraction with QIAmp DNA FFPE tissue kit (Qiagen, Inc., Valencia, CA). Quality and quantity of samples was assessed and improved by ethanol standard precipitation when necessary. Multiplex Ligation-dependent Probe Amplification (MLPA) was performed to determine the *EGFRvIII* form, analyzing the loss of exons 2 to 7. SALSA MLPA kit P105-C1 lot 1008 and ME024-A1 lot 0210 were used following manufacturer’s instructions (MRC-Holland, Amsterdam, The Netherlands). Fragments were separated by capillary electrophoresis in an ABI 310 Sequencer (Applied Biosystems, Inc, Foster City, CA) and data analysis was performed with the Coffalyser excel-based software (MRC-Holland).

### miRNA expression microarray analysis

Total RNA from frozen tissue of different groups was extracted using a mirVANA miRNA Isolation Kit (Ambion, Austin, TX, USA). The quality and integrity of total RNA was determined by capillary electrophoresis using the Bioanalyzer 2100 (Agilent Technologies, Santa Clara, CA, USA). Only RNA extracts with RNA integrity number (RIN) values ≥6 underwent a further analysis.

The GeneChip miRNA Array containing 46,228 probe sets representing 6703 microRNA sequences (71 organisms) from the Sanger miRNA database (V.11) and an additional 922 sequences of Human snoRNA and scaRNA from the Ensembl database and snoRNABase (Affymetrix, Santa Clara, CA, USA) was used for microarray analysis.

Microarray experiments were conducted according to manufacturer’s instructions. Briefly, 300 ng total RNA was labeled with FlashTag Biotin HSR RNA Labeling Kit from Genisphere. The labeling reaction was hybridized on the miRNA Array in Affymetrix Hybridization Oven 640 (Affymetrix) at 48°C for 18 h. The arrays were stained with Fluidics Station 450 using fluidics script FS450_0003 (Affymetrix), and then scanned on GeneChip Scanner 3000 7G (Affymetrix, Santa Clara, CA, USA). GeneChip Command Console Software supplied by Affymetrix was used to generate CEL files. Raw and processed microarray data together with relevant protocols and clinical data are available in the ArrayExpress database (www.ebi.ac.uk/arrayexpress) under accession number E-MEXP-2478.

Data files (.CEL) were analyzed and filtered using software Partek Genomic Suite 6.6 (Partek Inc., St. Louis, MO, USA). Input files were normalized with the robust multi-chip average (RMA) algorithm for gene array and further analyzed for data summarization, normalization and quality control by using the web-based miRNA QC Tool software (www.affymetrix.com). miRNA probe outliers were defined as per the manufacturer’s instructions (Affymetrix). We used non-parametric Kruskal-Wallis test at a significance level of p<0.05 to select miRNAs differentially expressed between the tumors with high level of *EGFR* amplification and those with no *EGFR* amplification.

For further identification of potential clusters based on microRNAs expression and related to *EGFR* amplification status we performed multivariate analysis. Projection to latent structures discriminant analysis (PLS-DA) was applied to obtain useful information from whole-genome miRNA expression profiling data. PLS-DA builds a regression model for maximum separation between pre-defined classes and variables responsible for class. PLS-DA is a classification technique that encompasses the properties of Partial Least Squares regression with the discrimination power of discriminant analysis. The main advantage of PLS-DA models is that the main sources of variability in the data are modeled by the so-called latent variables (LVs), and consequently, in their association with scores and loadings, allowing the visualization and understanding of different patterns and relationships in the data. Discriminant analysis was performed using the PLS Toolbox 6.7.1 statistical multivariate analysis library (Eigenvector Research, Inc. USA) in the Matlab R2008a environment (MathWorks, Inc., USA).

### Real-Time PCR analysis of mir-200c, CDH1, EGFR and ZEB1 expression

Expression levels of the selected miRNA (mir-200c) and mRNAs (CDH1, EGFR and ZEB1) were quantified using real-time reverse transcription-PCR (RT-PCR) analysis. 10 ng of total RNA was reverse transcribed with Taqman MicroRNA Reverse Transcription Kit (Applied Biosystems). The miRNA was amplified using commercially available Taqman probes (hsa-miR-200c, REF. 002300, Applied Biosystems). Taqman Universal Master Mix II (Applied Biosystems) was used for Real Time-PCR analysis.

Briefly, 300 ng total RNA was reverse transcribed with High Capacity RNA-to-cDNA Master Mix (Applied Biosystems). CDH1, EGFR and ZEB1 were amplified using commercially available Taqman probes Hs01023894_m1, Hs01076078_m1 and Hs00232783_m1 respectively (Applied Biosystems). The Taqman Gene Expression kit (Applied Biosystems) was used for Real Time-PCR analysis.

The relative differences in expression between groups were expressed using cycle threshold (Ct) values as follows. The Ct values were first normalized with RNU66 (REF: 001002) for mir-200c (REF: 002300) and ACTB (REF: Hs99999903_m1) for CDH1, EGFR and ZEB1 of the same sample. Statistical significance was tested with Student’s t test at a level of significance of p<0.05.

## Results

### Status of *EGFR*: correlation with clinicopathological parameters

**Figure 1:**
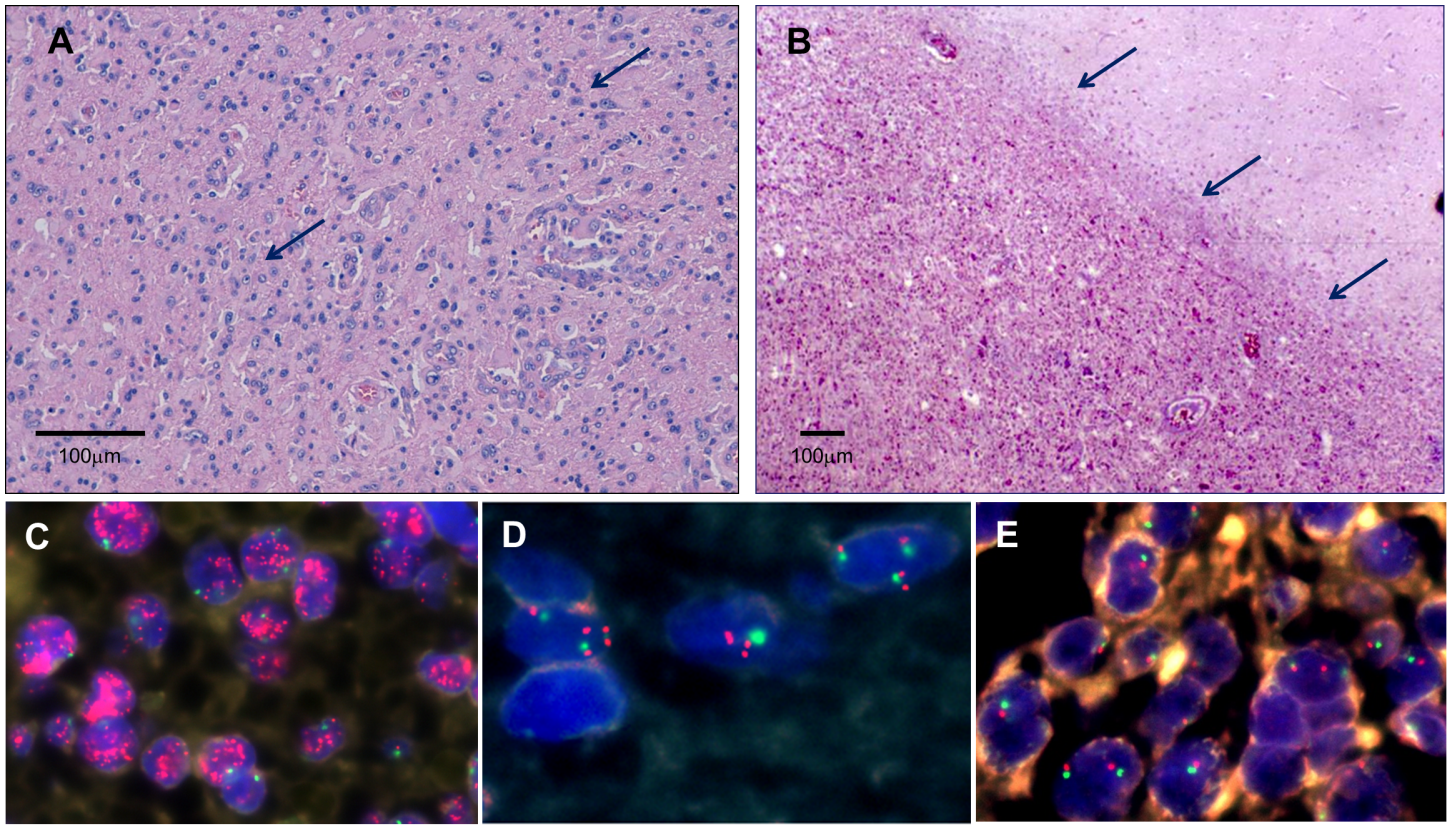
Patterns of infiltration in GBM and determination of *EGFR* amplification by fluorescence in situ hybridization with the dual probe for EGFR (red color) and centromere of chromosome 7 (green color). **A**. The cells tended to invade individually, giving a diffuse pattern (H&E, obj.x10). **B**. The infiltration was observed in groups of cells originating a nodular (or less-not diffuse) pattern showing a delimited front between the high density neoplastic cells and the peripheral nervous tissue (H&E, obj. x4). **C**. Tumor cells showing high-level amplification of the *EGFR*. **D**. Tumor cells with low-level amplification with other cells exhibiting a normal copy number of the *EGFR* gene. **E**. Cells without *EGFR* amplification.

Thirty primary GBMs were analyzed successfully by FISH in TMA. On basis of their *EGFR* status samples were categorized into three groups: (1) high level EGFR gene amplification (*EGFR*-ampH, 13 cases), (2) low level EGFR gene amplification (*EGFR*-ampL, 7 cases), and (3) no EGFR gene amplification (*EGFR*-Namp, 10 cases) (Figure 1C, 1D, 1E). The fraction of cells with highly amplified *EGFR* in group 1 was higher than 20%, with a large number of gene copies in each cell (more than 20 copies). The fraction of cells with amplified *EGFR* in group 2 ranged from 5% to 20% and showed a small number of *EGFR* copies in each cell (between 3 and 6 copies). Group 3 showed no *EGFR* amplification. Number of copies in each group was adjusted to paraffin sections with respect to whole cells in culture [7].

With the analysis by MLPA the mutant *EGFRvIII* form was found in 11 cases and the wild type *EGFR* was observed in 19 tumors. All cases with mutant *EGFRvIII* form, except one, were present in the group with high level of *EGFR* amplification. Only one case with *EGFRvIII* corresponded to the low level of *EGFR* amplification group (Table 1).

**Table 1:** Clinical, pathological and molecular findings in 30 cases of GBM.

Case	Age/Sex	Location	Size (cm)	Treatment	Survival (months)	Ki-67 (%)	EGFR expression	Pattern of infiltration	miR-200c 2-ΔΔCt	EGFR wild/mutant
1	72/F	F	6.0	RT+CH	8	14.4	3	Diffuse	0.38	EGFRvIII
2	63/F	T	7.0	RT+CH	11	8.5	3	Diffuse	0.06	EGFRvIII
3	69/M	T	6.0	NONE	6	10.4	3	Diffuse	0.26	EGFRvIII
4	48/M	T	5.3	RT+CH	9	10.0	3	Not established	0.19	EGFRvIII
5	55/F	CC	4.0	QT	4	14.0	3	Diffuse	0.60	EGFRvIII
6	58/F	T	6.0	RT+CH	23	21.8	3	Diffuse	0.12	EGFRvIII
7	61/M	T	8.0	RT+CH	11	1.6	2	Not established	0.26	EGFRvIII
8	59/F	P	1.5	RT+CH	27	4.5	3	Diffuse	na	EGFRwt
9	66/M	P	4.0	RT+CH	6	19.7	2	Not established	0.17	EGFRwt
10	55/M	P	6.0	RT+CH	10	3.8	2	Diffuse	0.46	EGFRvIII
11	69/M	T	6.0	RT+CH	13	50.0	2	Diffuse	0.29	EGFRwt
12	66/M	P	3.0	NONE	?	10.1	3	Diffuse	0.30	EGFRvIII
13	61/F	F	4.7	NONE	2	22.4	3	Diffuse	0.69	EGFRvIII
14	45/M	P	6.0	RT+CH	14	0.4	1	Not established	0.65	EGFRwt
15	24/M	T	2.5	NONE	?	13.0	1	Not established	1.44	EGFRwt
16	67/M	T	7.0	RT+CH	9	33.5	0	Diffuse	0.57	EGFRwt
17	73/M	P	5.0	RT+CH	3	19.9	0	Diffuse	1.33	EGFRwt
18	45/M	P	2.5	RT+CH	12	6.8	2	Diffuse	1.02	EGFRwt
19	60/M	P	4.0	NONE	5	0.8	1	Nodular	0.65	EGFRwt
20	72/F	P	3.7	RT+CH	30*	0.0	1	Nodular	1.95	EGFRvIII
21	66/F	F	6.0	NONE	2	5.1	1	Diffuse	1.49	EGFRwt
22	66/M	T	4.0	RT+CH	22	6.1	0	Diffuse	0.83	EGFRwt
23	63/M	F	4.0	RT+CH	36	23.0	0	Diffuse	0.63	EGFRwt
24	74/M	O	4.0	RT+CH	13	49.7	2	Nodular	1.21	EGFRwt
25	60/M	F	4.0	CH	18	3.0	1	Nodular	0.93	EGFRwt
26	55/M	F	6.6	CH	27	0.8	0	Nodular	1.02	EGFRwt
27	75/M	O	4.0	NONE	6	0.2	1	Diffuse	1.18	EGFRwt
28	74/M	F	6.7	RT+CH	1	10,5	1	Nodular	1.12	EGFRwt
29	63/F	P	8.0	RT	?	0.1	0	Diffuse	1.06	EGFRwt
30	67/M	P	3.5	RT+CH	50*	7.5	0	Diffuse	na	EGFRwt

Cases 1–13: High level of *EGFR* amplification; cases 14–20: Low level of *EGFR* amplification; cases 21–30: no *EGFR* amplification. Sex: male (M), female (F). Location: frontal (F), temporal (T), parietal (P), occipital (O), corpus callosum (CC). Treatment: radiotherapy (RT), chemotherapy (CH). Survival: all cases are exitus except (*) that were alive at the end of the present study and (?) that are cases with unknown evolution. (na): non-available. EGFRwt: EGFR wild type, EGFRvIII: EGFR mutant.

With respect to the clinical and histopathological characteristics (Table 1), in all three groups the incidence of tumor occurrence was higher in men than in women. The mean patient age was higher in group 3 **(**66 years) than in group 1 (61 years) and group 2 (55 years). Tumor localization was different in each group. In group 1 the tumor was located mainly in the temporal region, in group 2 the tumors were located predominantly in the parietal region and in group 3 predominant tumor location was in the frontal region. Tumor size was variable in each group. Primary treatment in all cases was surgery and adjuvant treatment predominated in all three groups (radiotherapy plus chemotherapy). Patient outcome in the different groups was as follows: in group 1 and 2 the mean survival was 10.8 and 12.1 months respectively, while in group 3 it was higher, 19.4 months.

Histologically, all tumors showed features of glioblastoma with pleomorphic astrocytic tumor cells, prominent microvascular proliferation and necrosis. In all cases GFAP expression was confirmed in the neoplastic cells. Histopathologically, two infiltration patterns were observed: diffuse infiltrative and nodular (or less-not diffuse pattern). We have considered these patterns when more than 75% of the peripheral tumor area could be analyzed. Diffuse infiltrative pattern showed a transition between the high density of neoplastic cells in the center of the tumor to diffuse and progressively decreased number of neoplastic cells in the periphery, showing an infiltration of the adjacent nervous tissue (Figure 1A). Nodular (or less-not diffuse) pattern shows a delimited front between the high density neoplastic cells and the peripheral nervous tissue. In this pattern the presence of isolated groups of neoplastic cells in perivascular spaces or in the neuropil has been observed, clearly separated from the tumor (Figure 1B). In this study, 16 cases have been considered as having a diffuse infiltrative pattern, 6 cases were considered to have a nodular pattern, while in 5 cases the infiltrative pattern could not be established. All the cases that showed a nodular-like pattern were tumors with no or low *EGFR* amplification.

The mean Ki-67 positive cells ratio showed a higher proliferation in group 1 (14.7%), followed by group 2 and 3 (10.6) (Table 1).

### Microarray analysis shows intermediate miRNA expression status for low *EGFR* amplification tumors

**Figure 2:**
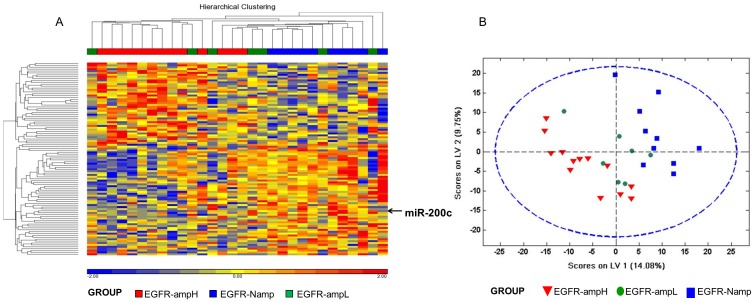
Microarray analysis of miRNA expression in 30 GBMs. **A.** Unsupervised hierarchical clustering of GBMs (horizontal dimension) and 102 miRNAs (vertical dimension) derived from a variance analysis. Over-expressed genes are represented in red and under-expressed ones in blue. **B.** The projection to latent structures discriminant analysis (PLS-DA) score scatter plot of the first 2 components. This PLSDA analysis discriminates the differences between GBM with high-level of *EGFR* amplification (n = 13) versus GBM non-amplified *EGFR* (n = 10). The symbols correspond as follows: red triangle, GMB with high level of amplified *EGFR* samples; blue square, non-amplified *EGFR* samples; green circle, samples with a low level of *EFGR* amplification.

We performed a highly restrictive analysis using Partek Genomic Suite v 6.6. We detected 119 small non-coding RNAs and within them 64 miRNAs whose levels changed significantly between samples of group 1 and group 3 (Table 2). An unsupervised hierarchical cluster analysis (HCA) was performed including these 119 small non-coding RNAs (Figure 2A). The heat map shows that group 2 samples were scattered between group 1 and group 3 samples. For better identification of global miRNA relationships among groups, a group 1 vs. group 3 discrimination model based on projection to latent structures discriminant analysis (PLS-DA) on whole-genome miRNA profiles obtained using Genechip miRNA Array was built. PLS-DA scores plot shows complete discrimination of the samples between these two groups. Then, group 2 samples were projected over the latent space of this discriminant model. In this space, samples from group 2 were located in the region between group 1 and group 3, suggesting that tumors with low *EGFR* amplification levels have a global miRNA expression profile intermediate between tumors with a high level of *EGFR* amplification and tumors without *EGFR* amplification (Figure 2B).

**Table 2:** miRNAs differentially expressed between *EGFR*-ampH and *EGFR*-Namp samples ordered by statistical significance (P value ≤0.05).

EGFR-ampH vs EGFR-Namp Probeset ID	P-value	Fold Change	EGFR-ampH vs EGFR-Namp Probeset ID	P-value	Fold Change
**Down-regulated**			**Up-regulated**		
hsa-miR-193a-3p_st	1.15E-03	−2.05	hsa-miR-320b_st	4.33E-03	1.49
hsa-miR-892b_st	1.26E-03	−2.75	hsa-miR-320a_st	5.25E-03	1.48
**hsa-miR-200c_st**	**2.37E-03**	−**3.84**	hsa-miR-320d_st	5.25E-03	1.50
hsa-miR-34c-3p_st	2.41E-03	−1.91	hsa-miR-320c_st	6.35E-03	1.42
**hsa-miR-138_st**	**2.91E-03**	−**3.47**	hsa-miR-1228_st	1.09E-02	1.66
hsa-miR-299-5p_st	2.91E-03	−2.34	hsa-miR-548f_st	1.09E-02	1.27
**hsa-miR-891a_st**	**3.55E-03**	−**6.89**	**hsa-miR-571_st**	**1.09E-02**	**1.22**
hsa-miR-200a_st	7.65E-03	−1.84	hsa-miR-106a-star_st	1.31E-02	1.48
hsa-miR-363_st	7.66E-03	−1.60	hsa-miR-496_st	1.84E-02	1.27
hsa-miR-342-5p_st	9.19E-03	−1.36	hsa-miR-877_st	1.84E-02	1.44
hsa-miR-569_st	9.19E-03	−1.41	hsa-miR-1292_st	2.55E-02	1.29
**hsa-miR-129-5p_st**	1.09E-02	−**2.99**	hsa-miR-675_st	2.99E-02	1.42
hsa-miR-342-3p_st	1.09E-02	−1.37	hsa-miR-876-3p_st	2.99E-02	1.29
**hsa-miR-379_st**	1.09E-02	−**3.56**	hsa-miR-32_st	3.49E-02	1.27
hsa-miR-491-5p_st	1.09E-02	−2.03	hsa-miR-340_st	3.49E-02	1.17
**hsa-miR-124_st**	1.31E-02	−**4.64**	hsa-miR-340-star_st	4.07E-02	1.13
**hsa-miR-128_st**	1.31E-02	−**2.54**	hsa-miR-1273_st	4.71E-02	1.25
hsa-miR-193a-5p_st	1.31E-02	−2.05	hsa-miR-549_st	4.71E-02	1.12
hsa-miR-132_st	1.84E-02	−1.61	hsa-miR-570_st	4.71E-02	1.22
hsa-miR-134_st	1.84E-02	−2.46			
hsa-miR-148a-star_st	1.84E-02	−1.41			
hsa-miR-586_st	1.84E-02	−1.28			
hsa-miR-409-3p_st	2.21E-02	−1.99			
hsa-miR-124-star_st	2.25E-02	−1.79			
hsa-miR-203_st	2.25E-02	−1.71			
hsa-miR-507_st	2.25E-02	−1.21			
hsa-miR-890_st	2.25E-02	−1.38			
hsa-miR-10a-star_st	2.99E-02	−1.41			
hsa-miR-129-3p_st	2.99E-02	−1.52			
hsa-miR-139-5p_st	2.99E-02	−2.38			
hsa-miR-217_st	2.99E-02	−2.78			
hsa-miR-377-star_st	2.99E-02	−1.72			
hsa-miR-127-3p_st	3.49E-02	−2.44			
**hsa-miR-382_st**	**3.49E-02**	−**2.66**			
hsa-miR-523-star_st	3.49E-02	−1.22			
hsa-miR-654-3p_st	3.49E-02	−1.83			
hsa-miR-375_st	4.07E-02	−1.41			
**hsa-miR-383_st**	**4.07E-02**	−**3.47**			
hsa-miR-886-3p_st	4.07E-02	−2.07			
hsa-miR-1262_st	4.71E-02	−1.26			
**hsa-miR-219-2-3p_st**	**4.71E-02**	−**3.09**			
hsa-miR-367_st	4.71E-02	−1.18			
hsa-miR-485-3p_st	4.71E-02	−1.97			
hsa-miR-517b_st	4.71E-02	−1.18			
hsa-miR-590-3p_st	4.71E-02	−1.14			

miRNAs in bold are common to PLSDA model.

### EGFR expression in tumors with different *EGFR* amplification levels

**Figure 3:**
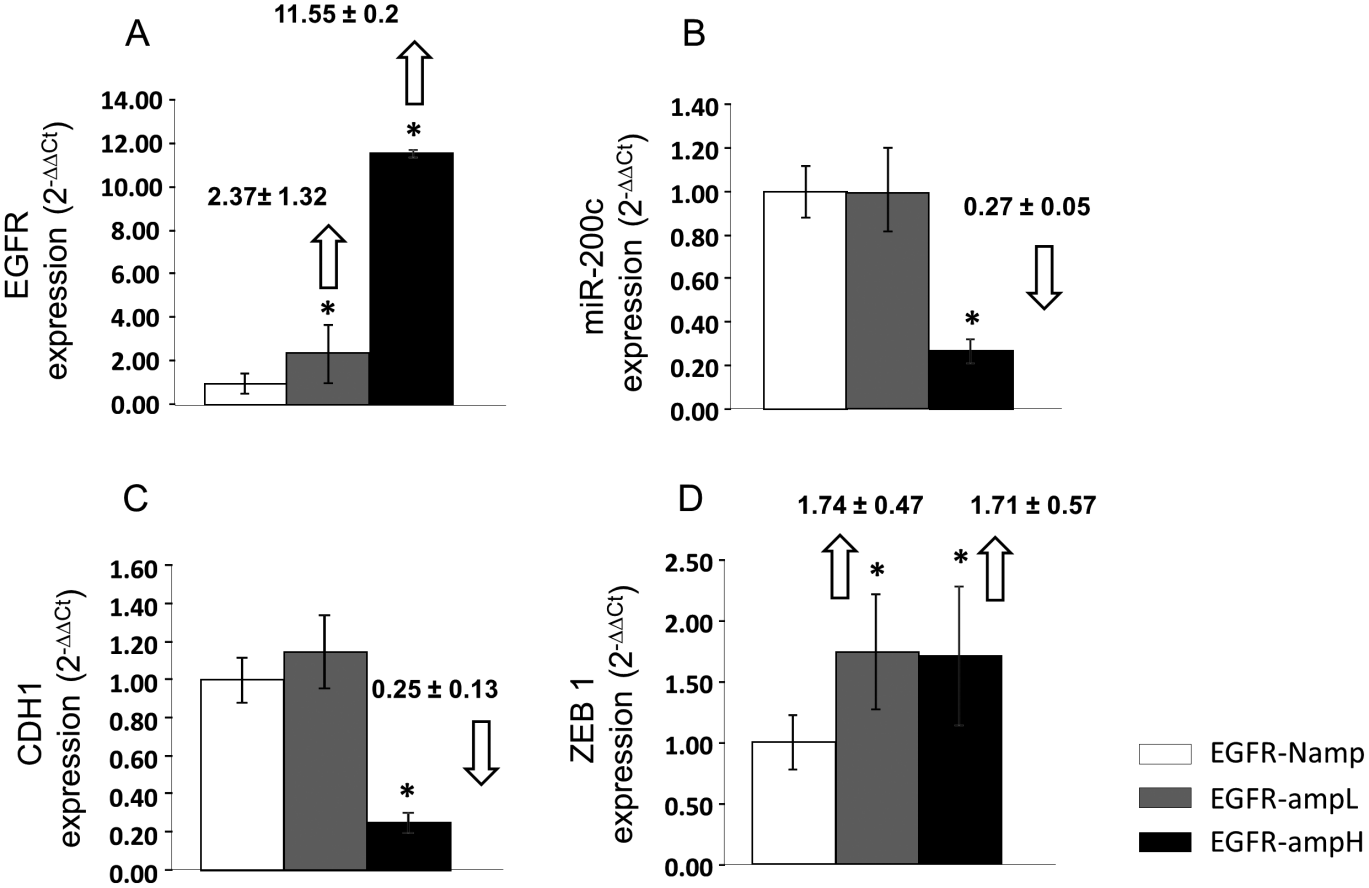
Real-Time RT-PCR analysis of EGFR, mir-200c, CDH1 and ZEB1 expression. Results are representative of 30 different samples. Changes in mRNA expression are reported as mean and standard error with respect to non-amplified *EGFR* group using the 2^−ΔΔCt^ method. Statistically significant expression changes (p<0.05) are marked with an *. **A.** EGFR mRNA expression in the GBM groups with different levels of *EGFR* amplification. The results were normalized to the actin housekeeping gene. **B.** miR-200c expression in tumor biopsies from the three studied groups. The results were normalized to the U66 housekeeping gene for miR-200c. **C.** CDH1 mRNA expression and **D**. ZEB1 mRNA expression in biopsies from the three studied groups. The results were normalized to the actin housekeeping gene.

EGFR mRNA expression was quantified by real-time RT-PCR. The tumors with a high level of *EGFR* amplification (group 1) showed more than a ten-fold increase in EGFR mRNA expression, while the tumors with a low level of *EGFR* amplification (group 2) showed a more than two-fold increase of EGFR mRNA expression, with respect to the tumors with non-amplified *EGFR* (group 3) (Figure 3A). With regard to EGFR protein expression analyzed by immunohistochemistry, all cases in group 1 showed over-expression, in group 2 the expression was variable, while in group 3 only one case showed over-expression (Table 1).

### miR-200c expression in tumors with different level of *EGFR* amplification and expression

Among the miRNAs differentially expressed between group 1 and group 3 samples, miR-200c exhibited very high statistical significance (Table 2). Analysis of the TCGA glioblastoma multiforme dataset [22], [23] found that miR-200c expression also shows a statistically significant decrease (p value<1e-5) in samples with high *EGFR* amplification in this dataset.

This result was validated by real-time RT-PCR expression quantification in the three groups (Figure 3B). We observed that the group 1 samples have statistically lower levels of miR-200c than the other groups. Samples in group 2 do not show statistically significant differences with respect to group 3 samples.

### Analysis of CDH1 and ZEB1 as possible targets of *EGFR* amplification

As previous studies have reported [19]–[21], the miR-200 family plays a role in a double-negative feedback loop with the ZEB family of transcription factors, which in turn suppress expression of E-cadherin (encoded by CDH1 gene). For further clarification of the potential role of miR-200c in GBM biology and to get some insight on the molecular mechanisms triggered by *EGFR* amplification, we also analyzed CDH1 and ZEB1 expression levels by real-time RT-PCR. Group 1 tumors show lower CDH1 expression levels than group 2 and group 3 tumors. The CDH1 expression levels for group 2 and group 3 samples did not exhibit statistically significant differences (Figure 3C). Conversely, ZEB1 mRNA expression levels were significantly lower in group 3 samples with respect to group 1 samples (Figure 3D). However, group 2 samples did not follow the same trend. Group 2 samples showed ZEB1 expression levels comparable to those detected in group 1 tumors. Analysis of the TCGA glioblastoma multiforme dataset [22], [23] found that ZEB1 expression also shows a statistically significant increase (p value<0.05) in samples with high *EGFR* amplification in this dataset.

## Discussion

Most patients affected by GBM present a recurrence of the disease at short periods of time. GBM is highly resistant to conventional therapies, including surgery. The highly invasive nature of these tumors makes it impossible to completely remove all tumor cells during surgical resection. Extensive infiltration of the surrounding brain tissue, which is an essential feature of GBMs, highly contributes to this deadly nature. In this infiltration, the tumor cells tend to invade individually or in small groups the surrounding tissue [24], [25]. GBMs in an orthotopic xenograft model can display two types of growth behavior: expansion of the tumor mass and formation of tumor nodules (NL-type) or highly diffuse single tumor cell infiltration (HD-type) [26]. Recent studies suggest that EGFR amplification and activation are correlated with invasive/non-angiogenic tumor growth [27].

In our study, most cases showed a highly diffuse growth pattern, as expected for highly aggressive tumors like GBM. However, all the cases that showed a nodular growth pattern-like were tumors with no or low *EGFR* amplification, included in group 2 or group 3 samples. Interestingly, previous studies did not find association between growth behavior patterns and mRNA expression levels of genes downstream of the *EGFR* pathway [26]. Our results suggest that this association may take place through the gene expression regulation by miR-200c instead.

Dysregulated *EGFR* signaling through genomic amplification, observed in about 35–70% of GBMs, is an important contributing event to the oncogenesis of high grade gliomas. Differences in the frequency of *EGFR* amplification found in the different studies are most probably due to the different methods used to determine *EGFR* amplification, such as Southern blot, polymerase chain reaction (PCR), and fluorescent *in situ* hybridization (FISH) [4]–[6]. In previous studies we described an *EGFR* amplification pattern that is shown by GBM tumors [7]. This *EGFR* amplification pattern suggests an early stage of *EGFR* amplification represented by cells with low levels of amplification. In the present study, GBM cases with different amplification status were analyzed. Cases with high *EGFR* amplification (group 1), which also exhibited over-expression of EGFR protein, showed increased Ki-67 proliferative index and reduced survival compared to cases that did not show *EGFR* amplification (group 3). The cases with low level of *EGFR* amplification (group 2) exhibited variable levels of protein expression, Ki-67 index similar to *EGFR* non-amplified tumors, but patient survival similar to high *EGFR* amplification tumors. All these parameters suggest that GBM with low *EGFR* amplification represent an intermediate state, which deserves further attention. These three different amplification statuses have been the reference for our study of miRNA expression.

The deregulation of miRNAs observed in several diseases, such as cancer, seems crucial for understanding their pathogenesis and has become an important factor to study principal regulatory systems and understand biological processes [12], [13]. Several studies have reported different miRNAs differentially expressed in GBMs when compared to low grade and/or anaplastic astrocytomas, suggesting an important role of altered miRNA expression in gliomagenesis and glioma progression [28]. In the search for molecular mechanisms relevant for GBM biological aggressiveness and regulated by *EGFR* amplification, we detected a group of miRNAs differentially expressed between samples with and without *EGFR* amplification. Our data show a clear relationship between *EGFR* amplification and miR-200c expression. In the tumors with high level of *EGFR* amplification, in which the EGFR expression is up-regulated, we observed that miR-200c expression levels are drastically diminished. To the best of our knowledge, miRNA-200c has not been described previously in GBM related to *EGFR* amplification. The miR-200 family regulates negatively EMT and cancer cell migration in many different epithelial tumors by targeting ZEB1 and ZEB2, which act as transcriptional repressors of E-cadherin [20], [21], [29]. Loss of miR-200c is associated with disease progression and poor outcome in different cell systems including breast cancer, bladder cancer, lung cancer and epithelial ovarian cancer [30]–[33].

Previous data indicate that the miR-200 family expression reduces motility and invasiveness while its inhibition increases migration capability [20], [29]. In our study, miR-200c under-expression in tumors with high *EGFR* amplification may be a potential mechanism for favoring more infiltrative patterns in GBM. In other types of tumors the activation of EGFR by its ligand (EGF) induces EMT. In the majority of anaplastic thyroid cancers EGFR is over-expressed and when activated by EGF a signaling cascade is activated that results in enhanced migration and invasiveness of thyroid cancer cells. EGF signaling correlated with the reduced expression of mir-200s and the re-expression of miR-200s abrogated the effects of EGF treatment [34]. In a bladder cancer cell line the stable expression of miR-200s increased E-cadherin levels, decreased expression of ZEB-1, ZEB-2, and cell migration, and increased sensitivity to EGFR-blocking agents [28]. Nevertheless, in cervical cancer, that presents an EGFR over-expression, EGF has been shown to be one of the most potent inducers of EMT and associated with cervical stromal invasion and nodal metastasis [35].

Cadherins regulate cellular adhesion within a tissue. The under-expression of cadherins produces an increase in cellular motility and in turn allows cancer cells to invade surrounding tissues [19], [36], [37]. Our data shows a positive correlation between miR-200c and CDH1. Tumors without *EGFR* amplification or low amplification showed the highest expression of CDH1. In addition, and related to the interplay among CDH1 and miR-200c, the group of tumors with a high levels of *EGFR* amplification showed high expression levels of ZEB1. These levels are lower in the group of tumors without *EGFR* amplification, which present a nodular infiltration pattern. In an intermediate situation, the group of tumors with a low level of *EGFR* amplification presents a high ZEB1 mRNA level, and also high levels of miR-200c and CDH1. This fact can be explained because ZEB1 protein expression is regulated by miR-200c at the translational level and therefore the consequent lack of the ZEB1 protein would impede the decrease of CDH1. An alternative explanation could involve GBM heterogeneity where different cellular populations may coexist in samples with different levels of *EGFR* amplification [38]. In these cases, cells with *EGFR* amplification may provide high levels of ZEB1 whereas cells with no EGFR amplification may provide high levels of CDH1.

A recent study shows that there is a strong link between the ZEB1 pathway and GBM invasion. The authors identified ZEB1 and other EMT-related factors in up to 50% of GBM patients. However, most of these factors were rather heterogeneous across the different samples. ZEB1 regulated by miR-200 was the most homogenous association described by the authors [39]. In our study, ZEB1 over-expression coupled with miR200c under-expression in tumors with high EGFR amplification may be a potential mechanism for favoring more infiltrative patterns in GBM.

Due to the astrocytic origin, the concept of straight EMT makes little sense in GBM, as these tumors rarely express E-cadherin [40], [41]. Nonetheless, expression of ZEB1 and other activators has been observed in GBM cells due to Wnt/β-catenin signaling [42], that increases cell motility in an epithelial-to-mesenchymal(-like) transition manner [43]. Recently, the effect of connective tissue growth factor (CTGF) has been described in glioma stem/tumor initiating cells (TIC/TSCs). CTGF activation of ZEB1 results in decreased CDH1 cadherin expression. This decreased expression of cadherin results in enhanced glioma cell invasion and migration [44].

### Conclusions

In summary, in this study we provide what is to our knowledge the first report of association between miR-200c and *EGFR* amplification pattern. Our results suggest that miR-200c may act as a potential regulator of GBM migration and invasion via targeting ZEB1. This study supports the existence of three groups of GBM considered according to the status of *EGFR* amplification with different levels of ZEB1 and miR-200c. Our findings show that miR-200c and CDH1 expression are down-regulated when *EGFR* is amplified (and over-expressed). The group with low-level amplification shows an intermediate behavior, with ZEB1 expression levels similar to the high-amplified *EGFR* group, but with CDH1 expression levels similar to the non-amplified *EGFR* group. These associations provide the basis for better understanding the aggressive nature of GBM and open new questions for further investigation.
